# Bridging language phenotypes, neural dynamics and gene regulation in autism

**DOI:** 10.1162/IMAG.a.1396

**Published:** 2026-10-16

**Authors:** Mariette Vinurel, Xiaoyue Wang, Djeser Kordon, Delphine Jochaut, Mario Speranza, Cedric Boeckx, Anne-Lise Giraud

**Affiliations:** Université Paris Cité, Institut Pasteur, AP-HP, Inserm, Fondation Pour l’Audition, Institut de l’Audition, IHU reConnect, Paris, France; EPS Barthelemy Durand, Etampes, France; Paris-Saclay University, UVSQ, Inserm, Center for Epidemiology and Population Health, Team DevPsy, Versailles, France; Department of Medical Psychology and Ethics, School of Basic Medical Sciences, Cheeloo College of Medicine, Shandong University, Jinan, Shandong, China; Fundamental Neuroscience Department, NCCR EvolvingLanguage, University of Geneva, Geneva, Switzerland; Service Universitaire de Psychiatrie de l’Enfant et de l’Adolescent, Centre Hospitalier de Versailles, Versailles, France; ICREA (Catalan Institute for Advanced Studies and Research), Barcelona, Spain; Universitat de Barcelona, Section of General Linguistics, Barcelona, Spain; Institute of Neurosciences, Universitat de Barcelona, Barcelona, Spain; Universitat de Barcelona Institute for Complex Systems (UBICS), Barcelona, Spain

**Keywords:** autism, speech processing, neural oscillations, genes, neuromodulation

## Abstract

Autism Spectrum Disorders (ASDs) affect approximately 1% of the global population, with prevalence rising over recent decades. Although ASDs are heritable (~70%), translating genetic findings into effective therapeutics remains challenging due to the vast number of implicated risk genes (>700) and the limited influence of individual genes on disease expression. Their collective impact and convergent expression patterns of small-effect genes provide insights into ASD-related neurobiological mechanisms. Current interventions remain symptom-focused and do not address underlying neurodevelopmental mechanisms. Emerging approaches in functional genomics offer new insights into ASD pathogenesis by exploring gene expression diversity, yet clinical applications remain distant. Language deficits are among the most frequent and disabling features of ASD, profoundly affecting individuals’ and families’ quality of life. Recent evidence from neuroimaging and electrophysiology studies suggests disrupted neural oscillatory dynamics are linked to language deficits in ASD. Several ASD-associated genes have been implicated in biological pathways involved in the regulation of brain oscillatory dynamics, suggesting a potential mechanistic link between genetic variation, altered brain rhythms, and language impairment. We propose a neurophysiological imaging genetics framework that leverages neural oscillatory profiles as endophenotypes for targeted investigation and as functional readouts of gene expression. Within this framework, neuromodulation is proposed as an experimental approach to investigate and potentially modulate altered oscillatory dynamics associated with speech communication deficits in ASD, while integrating oscillation-related ASD candidate genes into a symptom-oriented model of functional genomics. This approach shows how integrating genetic variation, non-invasive neuroimaging, and behavioral phenotypes can advance our understanding of ASD and inform targeted, mechanism-based interventions.

## 1 Introduction

Despite a broad consensus on its clinical definition (DSM-5; American Psychiatric Association, 2013) and core manifestations—including impairments in verbal and affective communication, repetitive behaviors, and restricted interests (Buch et al., 2023)—the therapeutic management of autism spectrum disorder (ASD) remains a major challenge. Current interventions primarily rely on behavioral therapies, such as Applied Behavior Analysis, and pharmacological approaches, including antipsychotic medications. While symptom-targeted behavioral interventions can substantially improve adaptive functioning, they may be constrained by underlying neurodevelopmental alterations that limit learning, generalization, and long-term outcomes. Targeting neural mechanisms, therefore, represents an important complementary strategy aimed at modifying the neurobiological substrate that shapes behavioral expression and responsiveness to environmental input.

Current limits in therapeutic interventions largely reflect the complex etiology of ASD and its heterogeneous genetic architecture, leading to a wide range of developmental trajectories. Hundreds of genes have been directly or indirectly associated with ASD risk (Bourgeron, 2016; Grove et al., 2019), yet variants with strong causal effects—such as those affecting SCN2A or SHANK3—account for only a small fraction of affected individuals. Although clinical trials are currently exploring targeted treatments for these rare monogenic or syndromic forms of ASD (Ioannidis et al., 2024), broadly applicable, evidence-based therapeutic options remain lacking.

Large-scale genetic studies aimed at refining ASD subtypes and their neurodevelopmental trajectories (Pagani et al., 2025; Satterstrom et al., 2020) hold promise for improving our understanding of disease mechanisms and informing therapeutic development. However, the highly polygenic nature of ASD—involving a large number of small effect common variants —substantially limits the direct translational relevance of these findings. We propose that restricting the set of candidate genes by focusing on those associated with specific phenotypic subgroups is a necessary step toward therapeutic progress.

In this context, the identification of intermediate neurobiological patterns represents a critical bridge between targeted genetic variation and clinically meaningful phenotypes that are both prevalent and central to functional outcome. Among these, verbal language development emerges as a particularly compelling target for mechanistically informed interventions. More than 50% of individuals with ASD experience verbal communication difficulties of varying severity, and up to 30% remain non- or minimally verbal (Anderson et al., 2007; Baio et al., 2018). Language abilities are a strong predictor of long-term functional outcomes and constitute a major concern for families (Tager-Flusberg & Kasari, 2013). In this framework, language deficits are not viewed merely as behavioral symptoms but as observable manifestations of underlying neurobiological mechanisms—particularly altered neural oscillatory dynamics—whose neural substrates are increasingly well characterized and may represent candidate endophenotypes and potential targets for mechanistically informed interventions.

Understanding spoken language relies on a quadruple challenge: comprehending what was just said while processing what is currently being said, anticipating what will come next, and planning a response. This sequence is managed through a well-orchestrated dynamic, ensuring that information flows at the right time and in the correct format throughout the language processing system (McClelland & Rogers, 2003). Key to this orchestration is the hierarchical coupling of specific neural oscillations that ensures the transformation of continuous speech into sequences of linguistic units through real-time integration of signals across timescales and processing stages (Bittar & Garner, 2024; Dimond et al., 2019; Giraud & Poeppel, 2012; Gnanateja et al., 2022; Meyer, 2018). Yet, in individuals with ASD, microstructural alterations of neural circuits are observed (Dimond et al., 2019), particularly in the minicolumns of the frontal and temporal lobes (Casanova et al., 2002). These structures are involved in the generation of neural oscillations (LaBerge & Kasevich, 2017) and their atypical organization is associated with disruptions in long-range connectivity (Casanova & Trippe, 2009), ultimately compromising the temporal integration of dynamic speech signals. In other words, language deficits in ASD may be linked to abnormal neural oscillatory activity. However, this pathophysiological hypothesis is not exclusive; rather it may represent one integrative mechanism through which diverse neurobiological alterations converge to affect language processing.

Language impairments in ASD appear early in development and are frequently reported retrospectively by adults, highlighting developmental timing as a critical dimension. Age-dependent changes in neural oscillatory dynamics and gene expression may, therefore, shape both the emergence and persistence of language deficits, potentially restricting the optimal windows for effective intervention.

## 2 Neural Oscillations: A Dynamic Neural Phenotype of Speech Processing

Neural oscillations arise from the synchronization of neural populations (Beppi et al., 2021; Buzsáki, 2001; Buzsáki & Vöröslakos, 2023) during specific sensory and cognitive operations. They result either from sustained interactions between excitatory and inhibitory neurons via chemical (Zou & Wang, 2016) or electrical synapses (Marder, 1998; Posłuszny, 2014; Yang et al., 2024). They can be measured from the scalp (EEG/MEG) or directly at the surface of the cortex (SEEG, ECoG). Commonly classified by frequency bands, they can combine (or couple) in specific ways depending on the cognitive functions they underpin (Hyafil et al., 2015; Lisman & Jensen, 2015; Siebenhühner et al., 2020). The range of neural oscillations and their hierarchical organization, with high frequencies reflecting fast/local processing and low frequencies coordinating slow/global integration, denote the timing of integration and information passing across functional modules. Akin to a stadium wave, where speed and uniformity depend on each section’s cohesiveness, neural oscillations indicate that the underlying structure is a unified network of micro functional modules (LaBerge & Kasevich, 2017). Disrupted oscillations thus indicate that functional modules cannot assemble to coherently integrate and pass the information (Hou et al., 2025). In speech and language processing, different families of neural oscillations coordinate hierarchical processes, enabling real-time encoding of speech at multiple linguistic levels (Kuhl et al., 2014; Poeppel & Monahan, 2011)—from phonemes to syllables, words, phrases, and discourse—and the sequential deployment of these elements during speech production (Fig. 1).

**Fig. 1.**
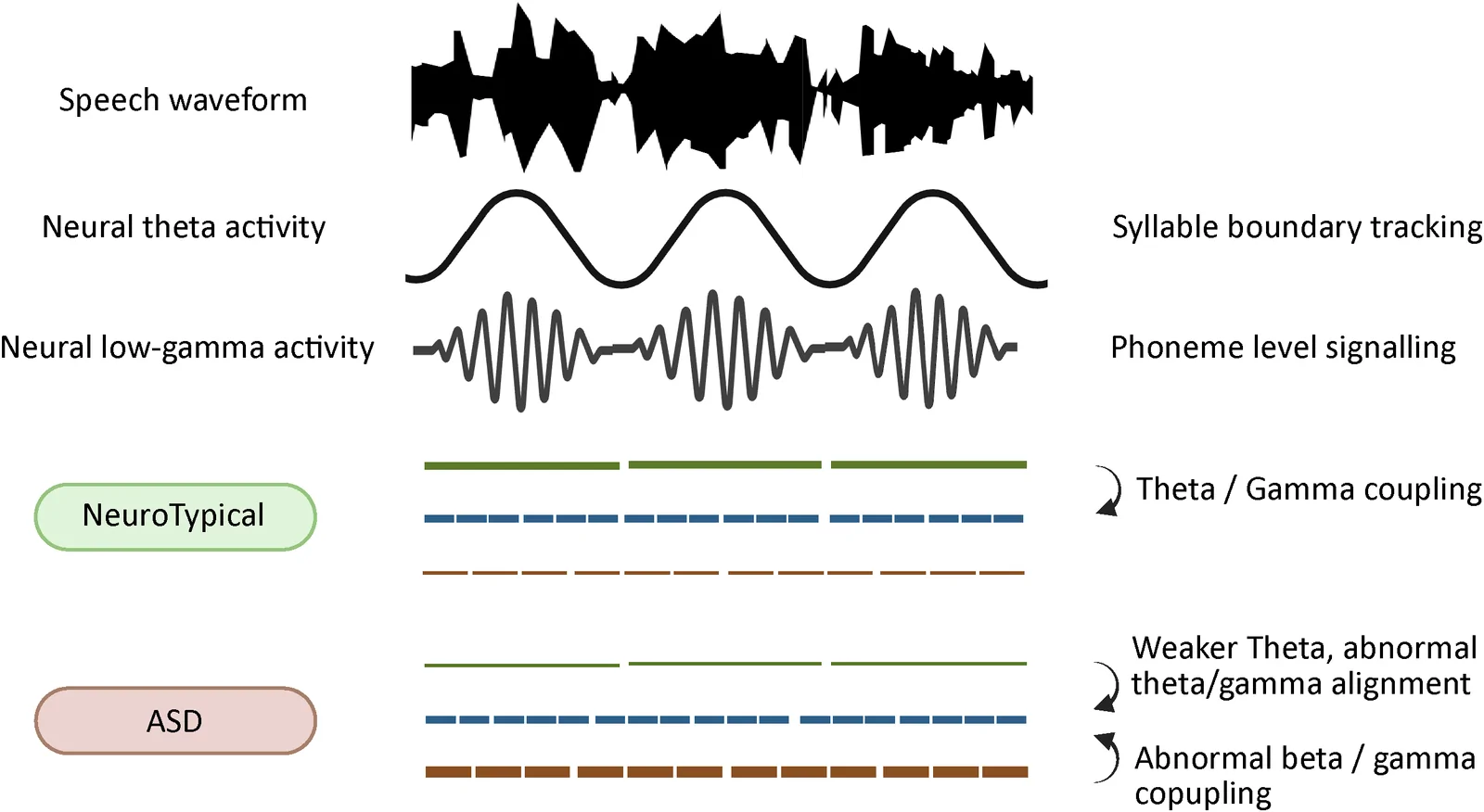
Basic oscillatory functional architecture of speech processing. In the neurotypical situation, the speech signal is marked by strong amplitude modulations corresponding to syllable boundaries. Theta oscillations track syllable boundaries and low-gamma oscillations encode phonemic cues within theta-syllable cycles, via theta-phase, gamma-amplitude coupling. In ASD, theta and gamma rhythms do not align, preventing phonemic encoding within syllables, and low-gamma activity is (abnormally) strongly coupled with the low-beta rhythm.

## 3 Syllable Segmentation, Phonemic Encoding, and Predictive Mechanisms

As the brain cannot process and integrate sensory information simultaneously and continuously, a critical step of natural speech processing is its segmentation into linguistic units, typically syllables. This step is facilitated by marked amplitude modulations in the speech acoustics signaling the syllabic structure. These sharp modulations, referred to as the speech envelope, occur between 4 and 8 Hz (Arnal et al., 2015), with an average of 5 Hz across all languages (Coupé et al., 2019; Poeppel & Assaneo, 2020). Syllable boundaries are tracked by a specific brain rhythm within the theta range (4–8 Hz), which flexibly adapts to the quasi-periodic nature of speech (He et al., 2023; Hyafil & Cernak, 2015; Hyafil et al., 2015). When listening to speech, the amplitude of theta oscillations in the auditory cortex increases, and their phase aligns with the syllabic rhythm and adapts to speech rate. Proto-syllable babbling begins around 4 months of age, and syllables eventually become the fundamental unit of speech and the building blocks for language acquisition. Concurrently, neural tracking of the rhythmic structure of speech emerges very early in development, with evidence for delta- and theta-band entrainment to speech already in newborns and strengthening during the first months of life (Attaheri et al., 2022; Ortiz-Barajas et al., 2021, 2023). In ASD children, theta-tracking is deficient and speech appears to be tracked by a slower brain rhythm, the delta oscillation (1–3 Hz) (Wang et al., 2023). In other words, children with ASD do not follow the syllable structure of speech but have a more global, stress and/or intonation-based initial segmentation, and these atypical mechanisms persist into adulthood (Jochaut et al., 2015).

Within syllables, phonemes are the smallest unit that can be both acoustically singled out and articulated. They elicit distinct neural responses, notably in the high- and low-gamma band of the EEG, around 25–40 Hz (Hoonhorst et al., 2009; Ou et al., 2018; Synigal et al., 2020). Phonemic encoding is regulated by the syllable-related theta activity, through a mechanism known as theta/gamma coupling (Arnal et al., 2015; Giraud & Poeppel, 2012; Jochaut et al., 2015; Leonard et al., 2024; Lizarazu et al., 2019; Orepic et al., 2023) (Fig. 1). In this process, theta cycles create windows of opportunity for encoding critical phonemic events, such as consonant bursts typically found at the onset of syllables (Giraud and Mamessier, 2020; Giraud & Poeppel, 2012; Gross et al., 2013), while low-gamma activity maps phonemic features.

Speech processing relies on bidirectional interactions between sensory bottom-up and top-down predictive processes (Fontolan et al., 2014; Hou et al., 2025). While bottom-up sensory analysis consists in extracting the syllabic structure and encoding phonetic information to access lexical features via theta tracking and theta/low-gamma coupling (Fontolan et al., 2014; Hovsepyan et al., 2023; Michalareas et al., 2016), top-down processing dynamically integrates contextual linguistic knowledge (Su et al., 2023) to anticipate speech content and timing (Gwilliams et al., 2022; Karunathilake et al., 2025; Niida et al., 2023) and prepare responses (Giordano et al., 2023; Karunathilake et al., 2024; Orepic et al., 2024). It is mediated by low-beta oscillations that propagate predictions from higher cognitive areas such as the prefrontal cortex back to lower-tier sensory regions. According to the predictive coding theory (Caucheteux et al., 2023; Friston & Kiebel, 2009; Rumelhart & McClelland, 1982), feed-forward bottom-up information corresponds to the mismatch between predicted and perceived signals, i.e. the prediction error. This dynamic bidirectional process enables parallel, temporally optimized analysis of speech features, with low-beta oscillations modulating the precision of predictions and gamma activity carrying sensory details (Hovsepyan et al., 2023; Pefkou et al., 2017).

In ASD individuals, weaker theta-syllable synchronization is accompanied by abnormal coupling of theta and low-gamma activity (Jochaut et al., 2015; Wang et al., 2023). This coupling is replaced by a coupling of low-gamma activity with top-down low-beta oscillation (15–20 Hz). These anomalies are predictive of the severity of autism symptoms in adults (Jochaut et al., 2015) and with language development in children (Wang et al., 2023). By compromising speech intelligibility, impaired syllabic segmentation (theta) and phonemic encoding (low-gamma) likely affect downstream steps of language symbolization and the ability to (re)produce connected speech from basic phonetic elements (Ghitza, 2011; Hovsepyan et al., 2020; Hyafil et al., 2015). These speech reception anomalies are consistent with reports from adults with ASD, describing profound difficulties in understanding spoken language during childhood (Jochaut et al., 2015; Wang et al., 2023).

The atypical beta/gamma activity observed in young children with ASD during speech-stream processing indicates abnormally strong coupling between predictive (beta) and sensory (low-gamma) mechanisms. This dysregulation may lead to overly rigid and stereotypical top-down predictions, reducing the brain’s sensitivity to acoustic variations in speech. Consequently, individuals struggle to update or refine their expectations based on incoming auditory information, leading to difficulties in decoding the dynamic features of spoken language. Inefficient predictions and error signaling are reflected in heightened neural responses to omitted sounds (van Laarhoven et al., 2020) and reduced habituation to self-generated stimuli (van Laarhoven et al., 2019) in participants with ASD. As another example, while neurotypical speakers of tonal languages suppress sensitivity to non-meaningful pitch variations, subjects with ASD struggle to contextualize meaningful tonal contrasts while detecting acoustical changes that are semantically irrelevant (Gomot et al., 2002; Sorokin et al., 2010; Wang et al., 2017; Yu et al., 2015).

To summarize, speech processing relies on exquisitely well-coordinated hierarchical neural oscillations: theta and gamma rhythms synergize via cross-frequency coupling to segment and decode speech input, while beta oscillations support top-down predictive mechanisms that guide perception and integrate sensory with contextual information. ASD individuals exhibit several neural anomalies in core aspects of speech processing, including reduced theta-syllable synchronization, impaired theta/low-gamma coupling, and abnormally strong low-beta/low-gamma coupling. Given the tightly interdependent nature of these oscillatory processes, disruptions in one component can cascade through the system, potentially leading to a variety of language deficits. As expanded in subsequent sections, such oscillatory signatures provide a relevant framework for exploring potential links with genetic variation and gene expression.

## 4 Bridging Symptoms, Neural Oscillations, and Functional Genomics

By considering oscillatory dynamics as functional intermediates that may reflect the combined influence of genetic variation and gene expression, we propose a methodological framework to investigate the biological underpinnings of speech impairments in ASD (Fig. 2). Within this framework, the oscillatory model of speech processing provides a scalable endophenotypic target that is both measurable using non-invasive neuroimaging methods such as EEG or MEG and experimentally modifiable through neuromodulation techniques, including neurofeedback and non-invasive brain stimulation (tES, TMS).

**Fig. 2.**
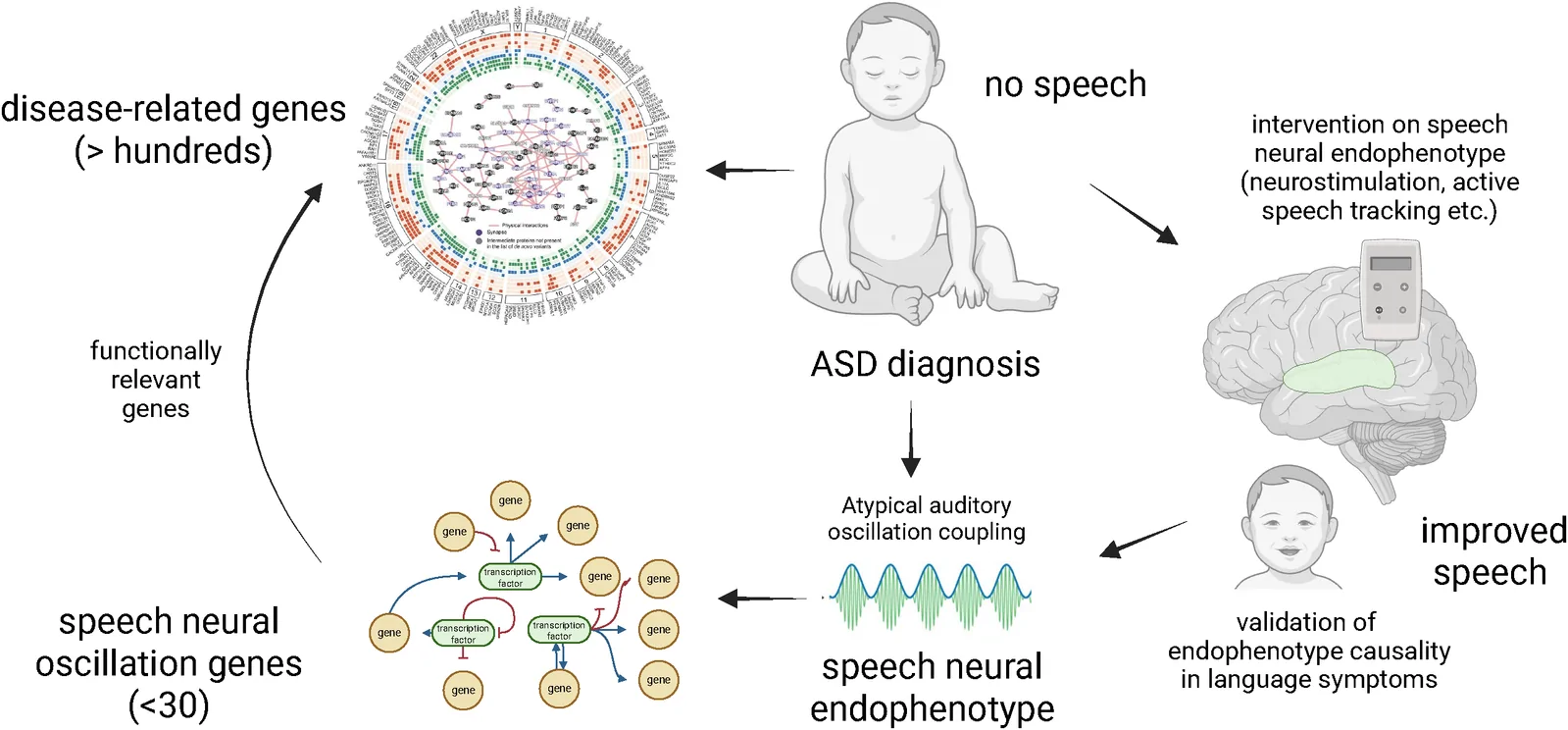
A methodological framework for exploring and treating speech communication difficulties in ASD. Hundreds of genes are associated with ASD based on a categorical diagnosis (affected/unaffected), making direct genetic interventions impracticable. Given that speech dysfunction constitutes a major source of disability in ASD, we use it as a starting point for a treatment-oriented and genomic research framework, in which functional genomics approaches allow the broad set of ASD-associated genes to be refined to a smaller subset of speech-related neural oscillation genes. These genes are hypothesized to converge on a speech-related neural endophenotype characterized by atypical auditory oscillatory coupling. Interventions acting on this endophenotype—including neuromodulation, active speech tracking, and pharmacological modulation— may help modulate oscillatory dynamics and could ultimately contribute to improving speech function, thereby providing an experimental framework to evaluate the contribution of neural oscillatory dynamics to language deficits in ASD. By focusing on functionally relevant genes identified through gene expression and isoform regulation in the developing brain, this framework enables more precise and biologically grounded investigations of ASD mechanisms and intervention strategies. The genome-wide map of autism susceptibility genes is credited to The Institut Pasteur. Created in BioRender. Mamessier Giraud, A. (2026) https://BioRender.com/3lf3kj7

In parallel, pharmacological approaches in ASD have historically relied on treatments that primarily address associated behavioral symptoms rather than core neurocognitive or mechanistically defined dysfunctions. For instance, currently approved treatments such as risperidone and aripiprazole remain largely symptomatic and non-specific (Hsia et al., 2014; McPheeters et al., 2011; Tomiyama et al., 2025).

More recently, pharmacological strategies have increasingly aimed to target specific molecular mechanisms, including synaptic transmission, excitation–inhibition balance, and neuromodulatory systems. These approaches are supported by a strong mechanistic rationale, as many ASD-related genes converge on molecular pathways involved in synaptic function and plasticity. For example, genes affecting GABAergic development and chloride transport (e.g., SLC12A2/NKCC1-related pathways) are directly relevant to the proposed action mechanism of bumetanide (Lemonnier et al., 2017); synaptic scaffolding and plasticity genes (e.g., SHANK3, FMR1) converge on mGluR-dependent signaling targeted by mGluR5 modulators (Berry-Kravis et al., 2016; Bhakar et al., 2012); and mTOR pathway dysregulation in syndromic ASD (e.g., TSC1/2) is directly targeted by everolimus (Curatolo et al., 2015).

However, despite this strong biological rationale, these targeted pharmacological approaches have yielded modest and highly variable clinical outcomes to date (Berry-Kravis et al., 2016; Curatolo et al., 2015; Sclabassi et al., 2025). A key limitation underlying these mixed results may be the persistent treatment of ASD as a unitary diagnostic entity, with insufficient consideration of underlying biological heterogeneity.

Importantly, many of the molecular pathways targeted by these emerging pharmacological strategies overlap with those implicated in oscillatory dysfunctions. Within this framework, oscillatory phenotypes may therefore provide a critical intermediate level of description linking gene expression, circuit dynamics, and behavior. They could serve not only as biomarkers but also as functional readouts of pharmacological target engagement at the circuit level, enabling the identification of biologically homogeneous subgroups more likely to respond to specific interventions.

Integrating such markers into clinical trial design may allow stratification based on circuit-level dysfunction rather than behavioural diagnosis alone, and may improve sensitivity to detect treatment effects in biologically defined subgroups. Ultimately, combining gene expression profiles, developmental timing, oscillatory biomarkers, and pharmacological target engagement may help bridge the gap between molecular mechanisms and clinical efficacy. This integrative approach holds promise for refining pharmacological targets and advancing a more individualized, mechanism-based treatment framework that could address part of the heterogeneity underlying the limited success of pharmacological interventions in ASD to date.

Neuromodulation approaches, particularly when implemented within stratified protocols incorporating developmental windows, oscillatory markers, and genetically informed profiles, may provide promising translational pathways for such precision interventions, potentially in combination with pharmacological modulation targeting shared molecular pathways.

## 5 Addressing Speech Deficits in ASD Through Neural Stimulation

A substantial body of evidence indicates that individuals with ASD exhibit atypical neural oscillatory activity across frequency bands implicated in sensory processing, language, and cognitive control. These oscillatory alterations have been documented using EEG and MEG and are thought to reflect disruptions in excitation–inhibition balance and large-scale functional connectivity. Several lines of evidence therefore suggest that oscillatory anomalies may be amenable to modulation through non-invasive neurostimulation techniques, including transcranial magnetic stimulation (TMS) and transcranial electrical stimulation (tES).

Importantly, strong causal evidence linking neurostimulation to improvements in language and auditory processing primarily derives from studies conducted outside the ASD population, notably in developmental dyslexia and acquired aphasia. In dyslexia, the application of tES over the left auditory cortex to normalize low-gamma oscillatory activity produces immediate and reproducible improvements in reading fluency and phonemic processing (Marchesotti et al., 2020). Similarly, in post-stroke aphasia, repeated TMS applied to language-related cortical regions has been associated with long-lasting improvements in language comprehension and reading abilities, persisting for up to two years following intervention (Georgiou & Kambanaros, 2022). Together, these findings indicate that oscillatory dynamics relevant to speech and language can be experimentally modulated, supporting the feasibility and mechanistic plausibility of oscillation-targeted interventions.

At the cellular and molecular levels, neuromodulation engages mechanisms directly relevant to neuroplasticity, including the upregulation of plasticity-related genes (Thomson et al., 2020), modulation of neurotransmitter systems and excitation–inhibition balance (Huerta & Volpe, 2009), and regulation of ion channel activity (Huerta & Volpe, 2009). These processes overlap with molecular pathways implicated in ASD-associated genetic risk, further supporting the link between oscillatory modulation and underlying neurodevelopmental mechanisms. In ASD, early neurostimulation studies suggest that TMS and tES can modulate cortical excitability, functional connectivity, and oscillatory patterns, with some studies reporting improvements in communication-related measures. Existing studies vary widely in stimulation protocols, targeted brain regions, outcome measures, and sample sizes, and robust conclusions regarding clinical efficacy will require confirmation through large-scale, well-controlled randomized clinical trials before clinical use can be justified (Wu et al., 2025).

Taken together, these findings support speech-related neural oscillations as promising candidate targets for neuromodulation-based approaches in ASD. Targeted neuromodulation aimed at correcting oscillatory anomalies may, therefore, offer a viable pathway for the development of future clinical trials focused on enhancing language skills.

Beyond their potential therapeutic value, non-invasive neurostimulation techniques provide a powerful experimental framework for testing causal relationships between neural oscillations and language performance. By assessing whether targeted modulation of oscillatory activity leads to measurable changes in speech perception or production, neuromodulation can be used to validate neural oscillations as intermediate endophenotypes (Qu et al., 2022; Willems et al., 2011). Such validation represents a critical step toward narrowing the genomic search space in ASD and linking genetic variation to functionally meaningful neurobiological mechanisms.

## 6 Toward a Genomics of Neural Oscillations in ASD

The capacity of neural networks to exhibit oscillatory behavior depends on a finite set of mechanisms involving ion channels, excitatory-inhibitory balance, and intracellular control of synaptic release which are controlled by various genes (Fig. 3) (den Hoed & Fisher, 2020; Khanna & Williams, 2021). Given that many ASD-associated genes are involved in synaptic plasticity (Bourgeron, 2015), there is an interesting overlap between genes linked to ASD and those involved in the regulation of neural oscillations. To identify these genes, we conducted a systematic literature review of genes implicated in neural oscillatory regulation (see Supplemental Materials), and cross-referenced the resulting gene set with the SFARI Gene database to identify ASD-associated candidates. An illustrative case is that of the SHANK3 gene and its link to low-gamma activity in response to auditory stimulation. In a patient diagnosed with language impairments and a rare partial SHANK3 duplication, auditory stimulation failed to elicit the typical 40-Hz auditory steady-state response, providing a plausible endophenotypic neural signature of a genetic defect (Neklyudova et al., 2021). As another example, abnormal theta-gamma coupling following auditory stimulation was detected in SynGap1 haploinsufficiency mouse models, establishing these sensory alterations as interesting translational biomarkers (Carreño-Muñoz et al., 2022).

**Fig. 3.**
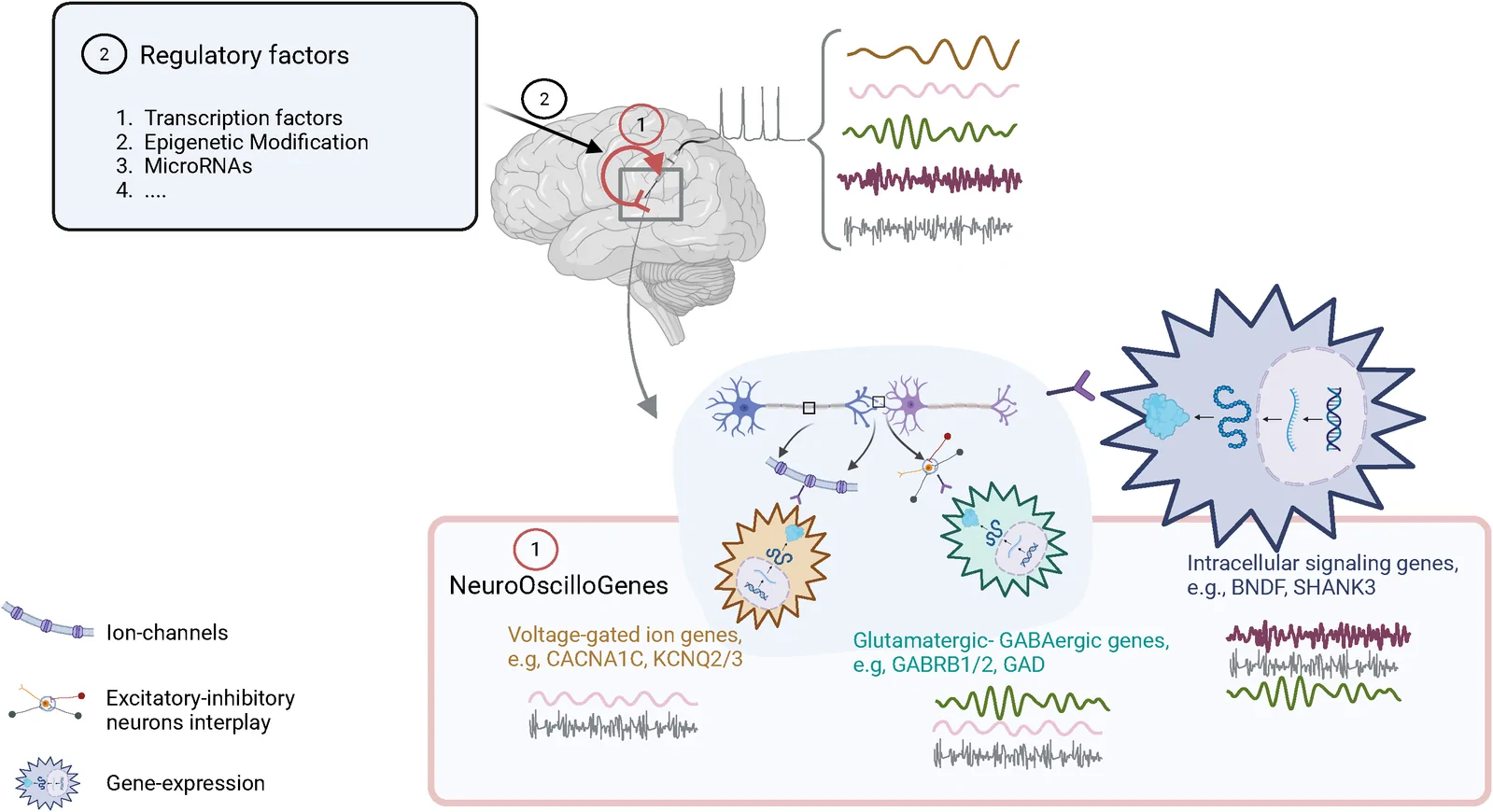
Different gene types involved in neural oscillations. 1. Protein-coding factors genes with variant neural oscillation activities. 2. Regulatory factors of gene expression which influence the protein-coding gene expression, subsequently impact neural oscillations in ASD. Conceptualized by X.W. and exported by M.V. Created in BioRender. Vinurel, M. (2026) https://BioRender.com/q76n834

Beyond gene-level effects, emerging transcriptomic evidence indicates that isoform-level regulation—including alternative splicing and differential transcript usage—can substantially modulate neuronal excitability, synaptic transmission, and circuit dynamics, without necessarily altering total gene expression levels; such isoform-specific regulatory mechanisms are shown to be particularly prominent during human brain development and are increasingly implicated in neurodevelopmental disorders, including ASD (Berto et al., 2018; Wen et al., 2024).

A strong argument to consider neural oscillations as critical phenotypes for genomic explorations in ASD is that the pool of genes controlling precisely these is far more restricted than that implicated in ASD as a global clinical entity. To illustrate the potential convergence between ASD risk genes and oscillatory control mechanisms, we conducted a structured literature search of studies published between 2003 and 2023 using the Scopus database, focusing on neural oscillations and their genetic underpinnings. This search yielded approximately 200 genes reported to be involved in the regulation of oscillatory activity across frequency bands. This gene set was subsequently cross-referenced with the SFARI Gene database (Abrahams et al., 2013), an evolving and curated resource cataloguing genes implicated in ASD susceptibility. From this intersection, we identified 22 genes classified as ASD risk candidates and reported to carry at least three independent de novo mutations in individuals with ASD. All 22 genes have documented roles in molecular or cellular processes known to influence neural oscillatory dynamics across different frequency bands (Fig. 4).

**Fig. 4.**
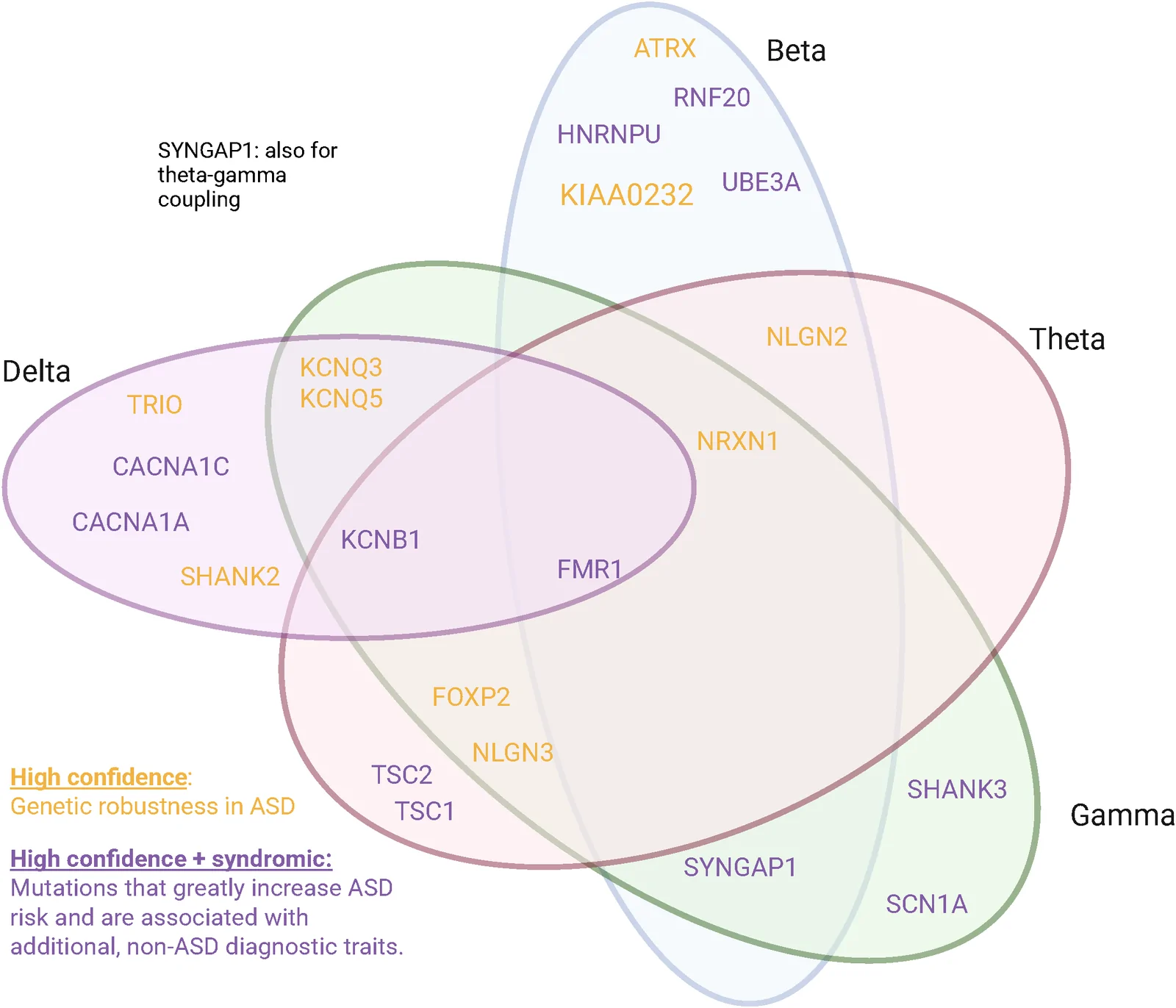
Genes of high confidence implicated in ASD are associated with different neural oscillations. Visualization of list of genes resulting from the cross-referencing of oscillation-related and ASD—associated gene pools based on a systematic literature review and on the SFARI Gene database respectively (Supplemental Materials). Genes are grouped by neural oscillations (Beta, Theta, Delta, Gamma) with each group represented by a color-code ellipse. Additionally, genes labeled in yellow font are high confidence ASD-associated genes with genetic robustness, while those in purple font are high confidence syndromic genes (associated with additional non-ASD traits). Note: SYNGAP1 is also implicated in theta-gamma coupling. Created in BioRender. Vinurel, M. (2026) https://BioRender.com/q64x710

For example, the CACNA1C and KCNQ2/KCNQ3 genes, which code for potassium and calcium channels, are critical in regulating neuronal excitability, timing of neuronal firing, and coherent oscillatory patterns (Klemz et al., 2022; Surti et al., 2005). These genes specifically influence theta (Tigaret et al., 2021) and gamma oscillations (Klemz et al., 2022), which, as seen above, are important for speech processing. Additionally, genes that regulate the balance between excitatory (glutamatergic) and inhibitory (GABAergic) neurotransmission, such as GABRB2 and GABRA1, also control gamma and theta oscillations, respectively, and GAD1, a gene that encodes an enzyme responsible for converting glutamate into GABA, is linked to both oscillations (Maillard et al., 2022; Tzilivaki et al., 2023). These and other candidate genes (Fig. 4) could, thus, be used for first an oscillatory-phenotype genetic mapping in ASD (leading for example to functionally relevant polygenic risk scores), and second for transcriptome-wide association studies in view of identifying possible therapeutic targets. The transcription mechanisms linked to specific oscillatory signatures are now being characterized across various cognitive tasks (Berto et al., 2018, 2021, 2022), confirming that it is possible to explore the genomics of specific cognitive operations. An important step will, thus, be to identify the most critical regulators in the reduced pool of ASD susceptibility genes involved in neural oscillatory behavior, but also those that may control them.

A last illustrative example is that of the XRN2 gene, which controls transcription termination and genome stability. Expressive quantitative trait loci (eQTLs) associated with ASD colocalize with regulatory regions that influence the expression of specific XRN2 isoforms (Kim et al., 2022; Wen et al., 2024; Zheng et al., 2024). Even modest shifts in the relative expression of gene isoforms—without any marked increase or decrease in total gene output—may contribute to various atypical patterns of brain development and neural functioning (Fernando et al., 2025). Such dysregulation could underlie some of the cognitive, behavioral, or sensory characteristics observed in ASD, and their huge diversity. Linking specific splicing patterns to oscillatory phenotypes thus appears as an interesting approach to target the molecular cascades that shape the control of cognitive operations by brain rhythms, opening new pathways for precision interventions, whether neuromodulatory initially or genomic in a longer-term perspective.

## 7 Conclusion

This perspective proposes that neural oscillations may constitute an important mechanistic bridge between genetic functional architecture and the language phenotype in ASD. This integrated approach proposes non-invasive neuromodulation techniques (e.g., neurofeedback, tES, TMS) as experimental approaches to modulate altered brain rhythms and evaluate their contribution to language symptoms related to syllable tracking, hierarchical embedding of speech elements, and speech prediction dynamics. If modulation of oscillatory dynamics were shown to improve receptive speech function, this would provide strong support for considering these neural signals as actionable endophenotypes for genetically informed investigations of ASD, focused on genes that control key neural mechanisms. Bridging low-dimensional gene expression profiles, oscillatory profiles, and speech-specific behavioral phenotypes will provide a valuable testbed for precision neuroscience in ASD.

## Supplementary Material

Supplementary Material

## Data Availability

No original data were generated or analyzed, and no code was developed or used for this article.
