# Supplementary material for: Bridging language phenotypes, neural dynamics and gene regulation in autism
Source: Imaging Neurosci (Camb). 2026 Oct 16;4:IMAG.a.1396. doi: 10.1162/IMAG.a.1396 (PMC13681050; doi:10.1162/IMAG.a.1396)
Supplement: Supplementary file 1 [file IMAG.a.1396_supp.pdf]

Supplemental Material

Method for identification of oscillation-related genes associated with ASD

Systematic literature review included search strategy development, eligibility criteria specification, screening and selection, data extraction, and analytical synthesis.

1. Search Strategy and Databases
The primary electronic database used was Scopus, selected for its comprehensive coverage of peer-reviewed literature in biomedical sciences, neuroscience, and psychiatry. The search was performed on January 15, 2024, and restricted to articles published between January 1, 2003, and December 31, 2025, in English. This timeframe was chosen to capture contemporary advances in genomics and neural oscillation research while ensuring methodological comparability across studies. A comprehensive search query was applied to the TITLE-ABS-KEY fields, combining terms related to genes (e.g., gene, genetic, mutation) and neural oscillations (e.g., brain wave, neural oscillation). Studies focusing exclusively on circadian rhythms were excluded. The search strategy was designed to retrieve articles within the core disciplines of biology, medicine, and neuroscience. No restrictions on study design were imposed at the initial search stage.

2. Inclusion and Exclusion Criteria
Studies were considered eligible if they reported direct or indirect associations between specific genes (in humans or animal models) and quantifiable neural oscillation metrics—such as power, coherence, phase-locking value, or cross-frequency coupling—measured via electrophysiological methods including electroencephalography (EEG), magnetoencephalography (MEG), intracranial EEG (iEEG), local field potentials (LFPs) in animal models, or biologically grounded computational modeling. Only articles published in English were included. Studies were excluded if they: (a) reported only behavioral or cognitive outcomes without neural oscillation measures; or (b) focused exclusively on non-neural oscillatory phenomena (e.g., cardiac or muscular rhythms).

3. Screening and Selection Process
The screening process was conducted in two steps. First, two independent reviewers assessed all retrieved records at the title and abstract level against the predefined eligibility criteria. Disagreements were resolved through discussion. Second, all potentially eligible articles underwent full-text assessment by the same review team. Reasons for exclusion at this stage—such as insufficient gene-specific data or absence of oscillation metrics—were documented.

4. Data Extraction
For each included study, the following information was extracted:
- Study characteristics: author(s), year, country, sample size, species (human, rodent, non-human primate), participant diagnosis or genetic status.
- Gene information: official gene symbol (HGNC nomenclature), genomic location, variant type (e.g., deletion, missense mutation, copy number variant), inheritance pattern
- Oscillation metrics: frequency band(s) analyzed (δ: 0.5–4 Hz; θ: 4–8 Hz; α: 8–12 Hz; β: 12–30 Hz; γ: 30–100+ Hz), metric type (e.g., power, coherence, phase-amplitude coupling), brain region or electrode site, and recording modality (EEG, MEG, LFP, etc.).
- Task context: resting state, sensory stimulation, cognitive task, or sleep. All extracted data were entered into a structured spreadsheet (Microsoft Excel) and independently cross-verified by a second reviewer.

5. Analytical Synthesis Approach

Due to substantial heterogeneity in methodologies, species, and outcome measures, a narrative synthesis was deemed more appropriate than a meta-analysis. Findings were grouped according to: (1) oscillation frequency band (δ, θ, α, β, γ); (2) gene functional category (synaptic adhesion, ion channel, transcription factor, signaling molecule); (3) task condition (resting vs. task-evoked); and (4) direction of effect (increase vs. decrease in power or coupling). Within each grouping, convergent findings across studies were highlighted and discrepancies noted. Particular emphasis was placed on genes with replicated associations across multiple independent cohorts or experimental models.

6. Gene Classification and Confidence Scoring

To assess the strength of evidence linking each gene to autism spectrum disorder (ASD), we referenced the SFARI Gene database (https://gene.sfari.org/, accessed December 2023). Genes were categorized as follows:

- High Confidence (Score 1): Strong evidence from multiple studies, including de novo loss-of-function variants and functional validation.
- Strong Candidate (Score 2): Multiple reports of rare variants with moderate functional evidence.
- Suggestive Evidence (Score 3): Limited but plausible association.
- Syndromic ASD genes: Genes associated with monogenic syndromes characterized by high ASD prevalence, classified separately but mapped to SFARI scores where available.
